# Advancing Lung Cancer Treatment: mRNA Therapeutics and Delivery Strategies

**DOI:** 10.1021/acs.nanolett.5c03826

**Published:** 2025-10-22

**Authors:** Alexey V. Yaremenko, Nadezhda A. Pechnikova, Chuang Liu

**Affiliations:** † Pulmonary Department, Oncology Unit, George Papanikolaou Hospital, School of Medicine, Aristotle University of Thessaloniki, Thessaloniki 54124, Greece; ‡ Section of Biomedical Engineering, School of Chemical Engineering, Aristotle University of Thessaloniki, Thessaloniki 54124, Greece; § Department of Neurosurgery, Brigham and Women’s Hospital, Harvard Medical School, Boston, Massachusetts 02115, United States

**Keywords:** mRNA therapeutics, lung cancer, lipid nanoparticles, cancer vaccines, genome editing

## Abstract

Lung cancer tops the list of the deadliest malignancies, consistently resisting conventional therapies and fueling the urgent pursuit of novel treatment strategies. Messenger RNA (mRNA) nanomedicines are rapidly expanding from pandemic vaccinology to oncology, but achieving efficient and targeted delivery to the lung continues to be a significant obstacle. This Mini-Review highlights advances that enable lung-focused mRNA therapeutics. We show extracellular and intracellular barriers, including mucus, surfactant, alveolar macrophages, and endosomal sequestration. We outline how ionizable lipids, polymer–lipid hybrids, extracellular-vesicle mimetics, and selective organ-targeting chemistry overcome these barriers. We overview the therapeutic payload spectrum, from multiepitope vaccines and antibody factories to tumor-suppressor restoration and *in vivo* gene editing, highlighting first-in-human data in lung cancer. We discuss persistent bottlenecks: off-target editing, cytokine toxicity, and manufacturing speed and propose design rules to accelerate translation. By integrating sequence-level mRNA design with precision nanocarriers, mRNA technology can benefit lung cancer therapy.

## Graphical abstract

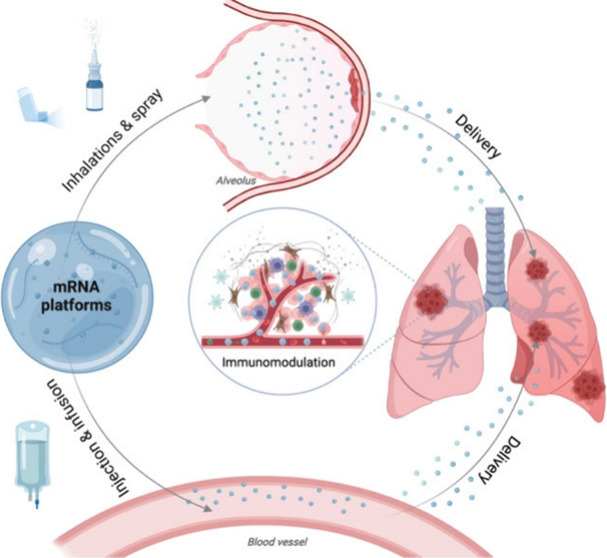

Despite continuing advances in tobacco-control and early detection efforts, lung cancer remains the most lethal malignancy worldwide. Globally, the GLOBOCAN 2022 update counts about 2.48 million new cases and 1.82 million deaths in 2022, placing lung cancer first for both incidence and mortality.^1^ In the United States alone, the American Cancer Society projects that approximately 226,000 new cases of people living with lung and bronchus cancer will be identified in 2025, with about 125,000 deaths, a toll that exceeds the combined deaths expected from colorectal, breast, and prostate cancers.^2^ Clinical outcomes of lung cancer remain poor, with survival rates plateauing below 25% for nonsmall-cell lung cancer (NSCLC) and 10% for small-cell lung cancer (SCLC), as metastatic progression and therapy resistance outpace even advanced targeted therapies.^3^ These sobering statistics highlight the critical need for novel therapeutic strategies that move beyond conventional cytotoxic chemotherapy, kinase inhibitors, and immune checkpoint blockade—the current cornerstones of lung cancer treatment.

Messenger RNA (mRNA) therapeutics have emerged as a potential platform capable of advancing lung cancer treatment. The rapid design-to-clinic trajectory and exceptional efficacy of the COVID-19 mRNA vaccines (BNT162b2 and mRNA-1273) demonstrated that lipid-nanoparticle (LNP)-encapsulated mRNA can be manufactured at a scale, elicit potent immunity, and remain safe in the recipients.^4–6^ Building on decades of foundational work in nucleoside modification, cap- and untranslated region (UTR)-optimization, and nanocarrier engineering, more than 120 clinical trials are now evaluating mRNA-based cancer immunotherapies across tumor types.^7,8^ Beyond vaccines, synthetic mRNAs can encode full-length antibodies, cytokines, tumor suppressors, or gene-editing components, enabling “programmable” pharmacology that is transient, nonintegrating, and readily personalized.^9,10^

However, mRNA therapeutics face both delivery hurdles and significant potential in lung cancer. For instance, mucus mesh, surfactant lipids, and alveolar macrophages constitute a formidable extracellular gauntlet, yet the organ’s vast epithelial surface and direct access via inhalation create unrivalled avenues for locoregional delivery.^11–15^ Advances in ionizable-lipid chemistry, polymer–lipid hybrids, and extracellular-vesicle mimetics are beginning to reconcile these opposing forces, enabling selective transfection of pulmonary immune and parenchymal cells while sparing liver and spleen.^14,16,17^

In this Mini-Review, we emphasize how rational mRNA design intersects with next-generation nanocarriers to surmount lung-specific biological barriers; highlight first-in-human data from lung-cancer vaccine, cytokine, and genome-editing trials; and map the translational inflection points most likely to convert preclinical promise into durable patient benefit. By integrating insights from molecular engineering, pulmonary drug delivery, and clinical oncology, we aim to show why and how mRNA nanomedicine could potentially redefine the therapeutic landscape for the world’s leading cause of cancer death.

## mRNA Delivery Platforms and Strategies to Overcome Lung Biological Barriers

Recent advances in mRNA technology, ranging from LNP vaccines to inhalable extracellular-vesicle formulations, have revitalized efforts to develop lung-directed treatments that can produce durable tumor control or even prevent relapse.^18,19^ Yet before any therapeutic message can act, it must run a gauntlet of pulmonary barriers that are far more complex than those encountered in liver or muscle, which were the target tissues for first-generation mRNA vaccines.^4,6^ The airways and alveoli of the lung impose far stricter cellular, extracellular, pharmacokinetic, and anatomical constraints (Figure 1).^11,13,20^ The extracellular barriers (Figure 1a), such as mucus and the mucociliary escalator, sweep particles toward the oropharynx. Additionally, surfactant lipids can destabilize cationic carriers, reactive oxygen species (ROS) generated by cigarette smoke or inflammation can fragment nucleic acids, and the secreted or systemic RNases, complement opsonins, and alveolar macrophages further reduce the administered dose.^11,13,21,22^ Even when nanoparticles reach type I/II pneumocytes, only 1% to 2 % of internalized RNA escapes the endosome^23^ (Figure 1b).

**Figure 1:**
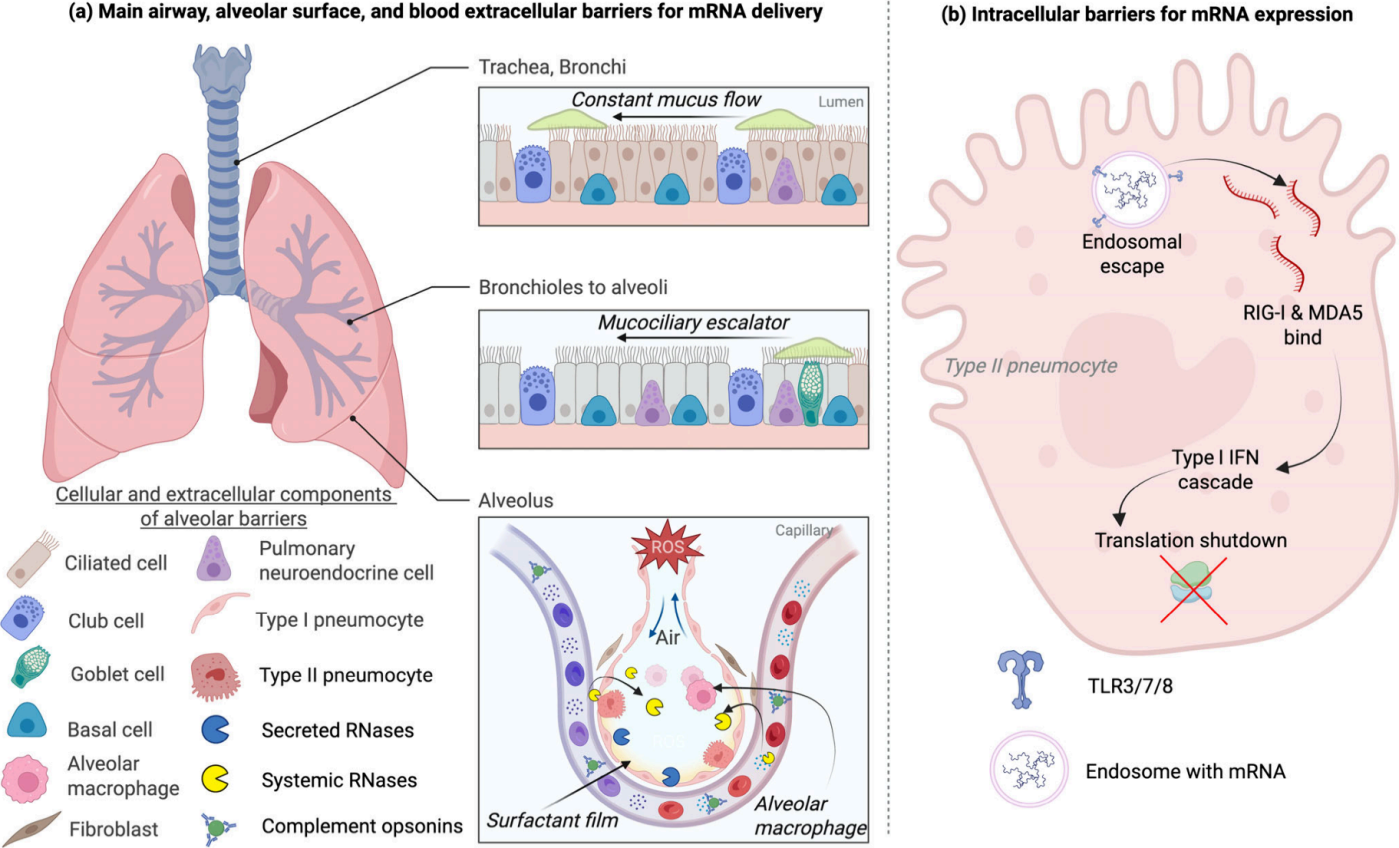
Barriers to effective mRNA delivery in the lung. (A) Airway/extracellular barriers. From trachea to alveoli, inhaled or blood-borne nanoparticles face mucus and mucociliary clearance, surfactant lipids, ROS, extracellular RNases, complement-mediated opsonization, and phagocytosis by alveolar macrophages. (B) Intracellular checkpoints. After uptake by epithelial cells (e.g., type II pneumocytes), only a small fraction of mRNA escapes endosomes to reach the cytosol; the remainder is detected by endosomal toll-like receptor (TLR) 3/7/8 or cytosolic retinoic-acid-inducible gene I (RIG-I)/melanoma differentiation-associated protein 5 (MDA5), initiating type I interferon (IFN) responses that suppress translation.

Despite extensive chemical and structural optimization, protein output from a single-shot LNP-mRNA dose remains inherently transient, as cells detect and clear exogenous mRNA. Cytosolic RNA sensors (RIG-I/MDA5) and endosomal TLR-7/8 recognize uncapped 5′-triphosphate or double-stranded motifs, triggering type-I IFN responses that suppress mRNA translation and activate RNases.^6,24,25^ A naked strand therefore persists in plasma for minutes and directs protein synthesis for only a few hours, insufficient for sustained cytokine, antibody, or clustered regularly interspaced short palindromic repeats (CRISPR) activity.^14,26^ Even “stealth” mRNAs with modified bases (e.g., N^1^-methylpseudouridine) and optimized UTRs blunt but do not abolish this innate sensing.^27^ In parallel, host mRNA decay pathways (e.g., deadenylation, decapping, and 5′ to 3′ exonuclease attack) inexorably degrade linear mRNA – unlike circular RNAs (circRNA) that resist exonucleases and thus sustain longer expression.^28^ Delivery adds further bottlenecks: typically less than 10% of LNP cargo escapes endosomes before lysosomal degradation,^29,30^ and lung-specific barriers (e.g., thick mucus, surfactant layers, and scavenging by alveolar macrophages) physically trap or clear particles.^25^ Thus, even with optimal 5′/3′ UTRs, caps, poly­(A) tails, and lipid formulations,^31^ mRNA-driven expression usually lasts only days. This brevity can limit applications needing prolonged protein (e.g., sustained tumor-suppressor expression or complete genome editing), whereas in vaccine settings, a short, intense antigen pulse is often sufficient to prime immunity.

To overcome these multilayered defenses, successful mRNA platforms now follow an integrated four-step choreography (Figure 2). (i) Molecular tailoring of the transcript, including designer caps, long poly­(A) tails, stabilizing UTRs, and immune-silent nucleosides, modulates innate immunity; (ii) architectural innovations such as self-amplifying RNA (saRNA) or trans-amplifying RNA (taRNA) and circRNA prolong cytosolic residency; (iii) nanoscale encapsulation with lipids, polymers or vesicle mimics shields the cargo, promotes endosomal escape and allows lung-tropic surface engineering; and (iv) route-of-administration choices (e.g., inhalation, intranasal, intratumoral or intravenous with lung-targeting chemistry, etc.) leverage, rather than battle, respiratory physiology.^11,13,20^ In comparison with first-generation systemic vaccines, these refinements collectively increase the proportion of the administered dose that reaches translation-competent cytosol in the lung, transforming mRNA from an academic curiosity into a realistic therapeutic for lung cancer.

**Figure 2:**
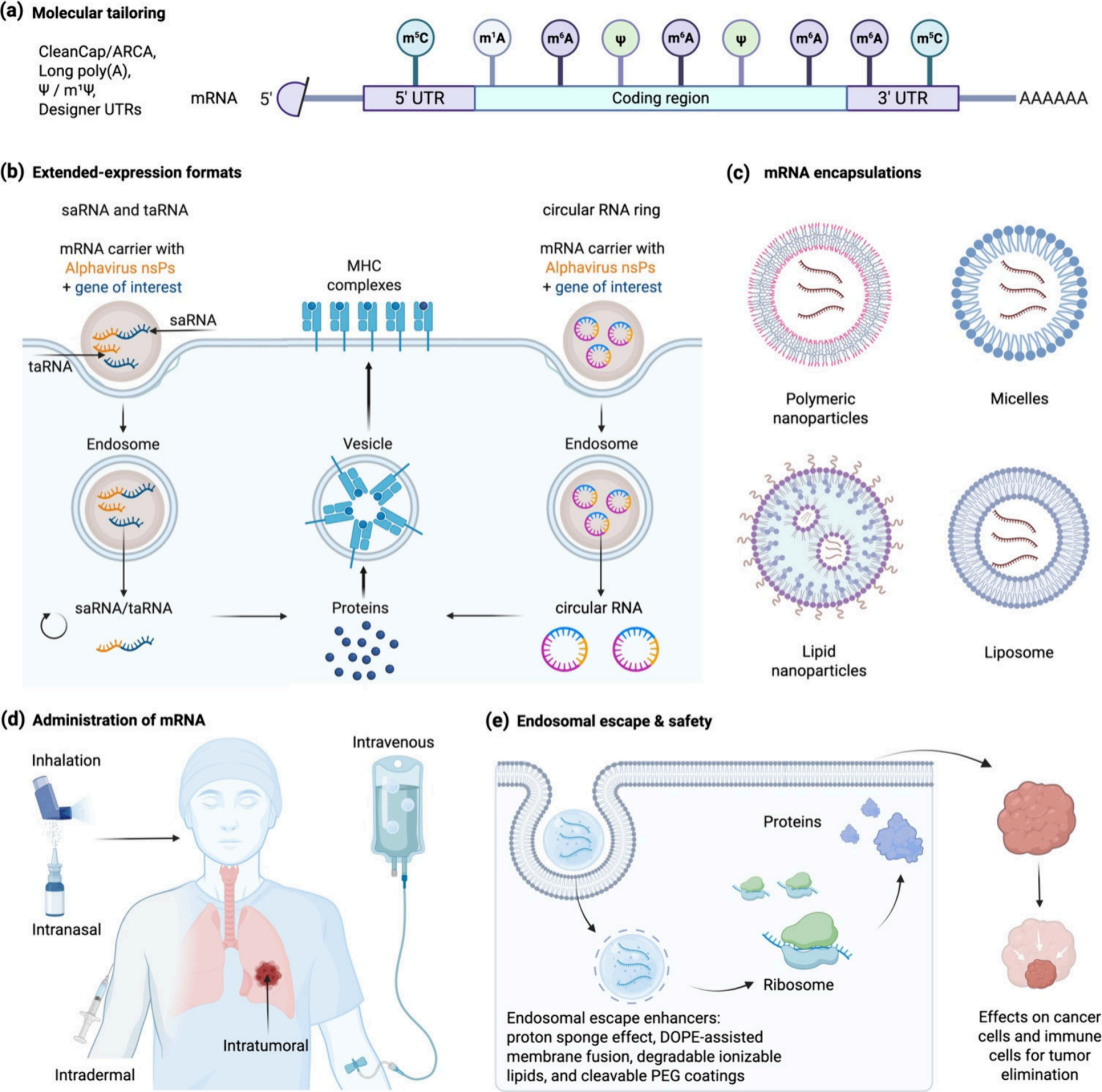
Integrated design principles for delivering therapeutic mRNA to the lung. (a) Molecular design the transcript through designer caps, long poly­(A) tails, stabilizing UTRs, and immune-silent nucleoside modifications; (b) extended-expression formats, self- and trans-amplifying RNA as well as circular RNA, sustain intracellular protein production; (c) diverse encapsulation platforms including polymeric nanoparticles, micelles, lipid nanoparticles and liposomes protect cargo and guide biodistribution; (d) multiple administration routes such as inhalation, intranasal, intradermal, intratumoral and intravenous delivery target the thorax with varying depth and scale; (e) endosomal-escape mechanisms and degradable carrier chemistries release the transcript for translation, ensuring effective and safe protein synthesis in lung tumor and immune cells.

Chemical engineering of the transcript provides the first layer of protection (Figure 2a). Co-transcriptional installation of Anti-Reverse Cap Analogues (ARCAs) or the newer “CleanCap” structures both stabilize the 5′ end against decapping enzymes and enhance eukaryotic initiation factor-4E recruitment, doubling translation efficiency in human bronchial epithelial cells.^14,32^ A 120- to 150-nucleotide poly­(A) tail recruits cytoplasmic poly­(A)-binding proteins and, together with the 5′ cap, folds the transcript into a closed-loop ribonucleoprotein that can reinitiate translation many times. When the tail shortens, the mRNA decays rapidly, so poly­(A) length is now tracked as a critical quality attribute during good manufacturing practice (GMP) production.^6,9^ UTRs are now mined via massively parallel reporter selections to eliminate adenylate-uridylate (AU)-rich decay elements and to introduce stabilizing motifs derived from α-globin or heat-shock transcripts, yielding order-of-magnitude gains in protein output.^9,10^ Optimizing the coding sequence to use codons that match the cell’s most abundant tRNAs and raising its guanine-cytosine (GC) content lets ribosomes move faster and reduces pauses during translation.^5,10^ Replacing the usual uridine bases with pseudouridine (Ψ) or N1-methyl-Ψ “camouflages” the mRNA, preventing the activation of innate immune sensors. As a result, translation continues, and the formulation can be dosed repeatedly without provoking systemic cytokine storms.^27,33^ These refinements have enabled milligram-scale production of clinical-grade mRNA for trials such as BI-1361849 (CV9202) in stage IV NSCLC.^34^ Beyond transcript stability, protein persistence can also be engineered at the sequence level. For example, a fusion protein expressed from mRNA encoding a zwitterionic EKP polypeptide (glutamate-lysine-proline repeats) fused to the C-terminus of the target protein markedly prolongs its serum half-life compared with the unfused protein.^35^ This approach produces a fusion protein without the need for postproduction modifications such as the covalent attachment of polyethylene glycol (PEG) to the protein, providing an mRNA-encoded alternative for prolonging protein half-life.

However, despite extensive optimization, protein expression from nonreplicating linear mRNA remains transient, typically peaking 12 to 24 h after delivery, declining sharply over the next 48 to 72 h, and returning to near-basal levels within approximately 1 week *in vivo*, including after intramuscular injection.^23,26,27^ Although this expression window is sufficient to prime an immune response in vaccination settings, it is inadequate for applications that require sustained protein output, such as antibody replacement, tumor-suppressor restoration, or CRISPR-based genome editing, where week-long production is preferable. Three architectural innovations are addressing that limitation (Figure 2b). For instance, saRNA and its two-component version, taRNA, carry alphavirus replicase genes, so the payload can copy itself inside the cell. A single intranasal dose of only 0.5 μg of saRNA has already protected ferrets against influenza, and similar constructs are now entering oncology trials at about 30 μg per patient.^36,37^ Additionally, covalently closed circRNAs lack the free ends that exonucleases normally degrade, so they persist much longer than linear strands. Using internal ribosome entry sites, they initiate cap-independent translation and can maintain neutralizing-antibody production in mice for several months—an exposure window that makes them appealing for chronic cancer therapy.^38^ As an example, recent work shows that therapeutic circRNAs can be selectively back-spliced, efficiently loaded into extracellular vesicles, and delivered *in vivo*, where they drive sustained protein production and even enable gene editing, underscoring the synergy between circRNA stability and vesicle targeting.^39^ The promise of these longer-lived formats is particularly compelling for lung tumors that reside behind multiple delivery barriers and may receive only small particle doses at each administration.

To protect mRNAs from degradation and achieve long-lasting expression, physical encapsulation is still critical (Figure 2c). LNP, comprising an ionizable amine-bearing lipid, helper phospholipid, cholesterol, and a PEG-lipid, remains the most clinically validated carrier class.^5^ At acidic pH, they condense and protect mRNA; at neutral pH, they shed charge to minimize complement activation, thereby permitting repeated intravenous dosing.^10,20^ The helper lipids (e.g., 1,2-distearoyl-*sn*-glycero-3-phosphocholine, DSPC) promote endosomal fusion, whereas cholesterol modulates bilayer rigidity and, when oxidized, can even retarget particles from hepatocytes to endothelial or pulmonary cells.^20^ Cationic lipoplexes based on N-[1-(2,3-dioleyloxy)­propyl]-N,N,N-trimethylammonium chloride (DOTMA), or 1,2-dioleoyl-3-trimethylammonium-propane (DOTAP), achieve high encapsulation efficiency. By simply adjusting the cationic lipoplexes to RNA molar ratio, researchers have switched the organ tropism of intravenously injected LNP from spleen to lung – an elegant demonstration of how surface charge steers the serum-protein corona and ultimately biodistribution.^40^ Polymer–lipid hybrids, ionizable dendrimers, calcium-phosphate cores, and shear-thinning hydrogels each offer alternative balances of biodegradability, endosomal escape, and thermal stability, and many can be lyophilized for room-temperature storage – an advantage for global lung-cancer trials that often recruit at community hospitals in low- and middle-income countries.^5,8,9,20^ More recently, extracellular-vesicle-mimetic carriers generated by “cellular nanoporation” have cloaked mRNA inside native membrane proteins that avoid phagocytic clearance and preferentially accumulate in inflamed lung tissue.^22^ Notably, engineering leukocyte-derived extracellular vesicles with retrovirus-like activity-regulated cytoskeleton-associated protein (Arc) capsid proteins greatly boosted mRNA loading and even enabled extracellular vesicles to cross restrictive biological barriers such as the blood-brain barrier. This technology illustrates how rational extracellular vesicle design can further extend the reach of vesicle-based mRNA delivery.^41^

Although intravenous infusion is the most mature route, systemically administered LNP must still traverse a gauntlet (Figure 2d): opsonization by serum proteins, Kupffer-cell filtration, extravasation through fenestrated but tumor-compressed pulmonary vasculature, and finally infiltration of a stiff extracellular matrix rich in hyaluronan and collagen.^12^ The selective organ-targeting (SORT) concept offers a solution. By doping standard four-lipid LNP with 10–40 mol % quaternary-amine lipids such as DOTAP, mRNA LNP can be predominantly rerouted to lung epithelial and endothelial cells after intravenous injection. This technology enables CRISPR homology-directed repair in cystic-fibrosis mouse lungs and demonstrates gene editing efficiencies unattainable with liver-tropic formulations.^42^ Translating SORT chemistry to oncology is straightforward: employing guide RNA targeting driver oncogenes (e.g., KRAS-G12C) or resistance mediators (e.g., KEAP1) could potentially rewrite tumor genomes *in situ*.^38^

Another way to overcome systemic hurdles could be local administration (Figure 2d). Direct intratumoral injection of lipid-packaged cytokine mRNAs (*interleukin-*23-, *interleukin-*36-, and *tumor necrosis factor ligand superfamily member*4-mRNA) has produced systemic antitumor immunity in multiple preclinical lung-cancer models and is now in first-in-human testing (NCT03739931), but repeated bronchoscopic injections are impractical for peripheral nodules or disseminated metastases.^43^ Alternatively, implantable biomaterial depots can confine and sustain local release of mRNA or immunomodulators in the thorax, potentially increasing efficacy while limiting systemic exposure.^44^ Also, aerosolized delivery is therefore garnering intense attention as an alternative noninvasive administration route.^13,45^ For example, an inhalable hyaluronic-acid/LNP engineered to cotarget lung tumor cells and inflammatory macrophages achieved high local uptake of *tumor protein p*53 (*p*53)-mRNA after aerosol dosing and triggered robust antitumor immunity in orthotopic lung-cancer models.^46^ Additionally, to overcome limitations related to the mucus macrophage-rich epithelium, the ionizable LNP morphology and PEG density were optimized to survive upon nebulization shear forces. The results demonstrated that inhaled particles, expressing a neutralizing antibody, reached distal alveoli more efficiently than an equivalent intravenous dose.^21^ Building on this, charge-assisted-stabilized LNP incorporating a peptide-lipid preserved over 40 % of particles after nebulization (versus about 17 % for PEG-LNP) and delivered approximately 7-fold higher mRNA levels to the lung, resulting in markedly stronger mucosal and systemic immune responses *in vivo*.^47^ A follow-up study took the approach a step further. *Interleukin-*12 (*IL-*12)-mRNA was packed into engineered extracellular vesicles small enough for nose-only inhalation. The vesicles deposited deeply throughout the lung caused regression of orthotopic LL/2 (mouse lewis lung carcinoma cell line) tumors, and even produced abscopal immunity against lesions in the opposite lung.^22^ Complementing these findings, lung-derived exosomes processed into a room-temperature-stable dry powder carried spike-mRNA deep into bronchioles and alveoli after inhalation, eliciting stronger mucosal IgA and systemic IgG than matched liposomal powders.^48^ Consistently, nebulized lung-sourced exosomes surpassed PEG-LNP for pulmonary mRNA delivery, showing superior distribution and retention across bronchiolar and alveolar regions.^49^ These studies highlight the double dividend of pulmonary delivery: elevated local concentration at the tumor site and reduced systemic exposure, thereby expanding the therapeutic window for potent immunostimulatory cytokines.

Nevertheless, there are obstacles to delivering an mRNA LNP via inhalation. First, LNP must penetrate a viscoelastic mucus mesh with pore sizes of 100 to 500 nm, dissolve through the surfactant film lining alveoli, and escape rapid engulfment by alveolar macrophages that clear about 85% of deposited nanoparticles within 24 h.^50,51^ Second, surface PEGylation of LNP can mitigate muco-adhesion but risks anti-PEG antibody formation during chronic therapy. To retain effective mucus penetration while minimizing this immunogenic risk, ongoing studies are testing zwitterionic surface chemistries and adding mucolytic excipients such as N-acetylcysteine.^15^ In addition, ultrasmall hydrodynamic diameters (less than 60 nm) navigate mucus pores more efficiently yet carry less mRNA. Also, modular “nanogel-in-nanoparticle” carriers, where a sturdier outer shell protects the cargo, then dissolves after crossing airway mucus to release much smaller, soft nanogel cores that diffuse more easily through the mucus mesh and reach epithelial cells.^50,52^ Furthermore, endosomal escape remains the final bottleneck: only 1–2% of internalized mRNA typically reaches the cytosol.^23^ To overcome these obstacles, adding ionizable lipids that stay neutral in the bloodstream but become positively charged inside the acidic endosome can improve mRNA endosomal escape; the resulting electrostatic stress destabilizes the endosomal membrane and promotes fusion with the nanoparticle. Conical “helper” lipids such as 1,2-dioleoyl-*sn*-glycero-3-phosphoethanolamine (DOPE) support this process by lowering the energy barrier for the membrane to adopt nonbilayer (hexagonal) shapes, which further speeds mRNA release into the cytosol (Figure 2e). Thus, grafting pH-responsive imidazole or histidine groups onto the ionizable lipid amplifies the “proton-sponge” effect: the side chains become protonated inside the acidic endosome, draw in counterions and water, and help rupture the membrane. The most effective chemistries are now identified by pooling thousands of DNA-barcoded lipids, delivering them *in vivo*, and then sequencing the barcodes that accumulate in lung cells – an approach that simultaneously pinpoints optimal lipid structures and the host genes that govern nanoparticle uptake.^53^

Across the current landscape, the approaches with the clearest momentum for lung delivery share a common logic: preserve particle integrity through aerosolization, raise the effective intracellular dose in airway and alveolar cells, and minimize systemic exposure. Aerosol-robust LNP that withstands nebulization shear and traverse airway mucus have repeatedly shown superior postnebulization recovery and higher pulmonary mRNA levels compared with conventional PEG-LNP, resulting in stronger local expression and mucosal/systemic immunity at lower doses.^21,47^ These gains reflect improved particle stability in the aerosol stream, reduced muco-adhesion, and facilitated endosomal escape in the acidifying endosome.^23,54^ In parallel, extracellular-vesicle mimetics delivered by inhalation distribute broadly across bronchiolar and alveolar regions, cloak cargo in native membrane proteins that evade rapid macrophage clearance, and can drive antitumor efficacy while curbing off-target exposure.^22,48,49^ For systemic dosing, SORT chemistry and rational ionizable-lipid design can reroute LNP from hepatocytes to pulmonary epithelial and endothelial cells and improve on-target transfection/editing in the lung,^38,42,55–57^ with performance gains arising from tuned ApoE adsorption, headgroup p*K*_a_, and degradable linker scaffolds that promote timely release. Critically, pooled *in vivo* barcoded discovery, where each candidate LNP carries a unique DNA barcode and barcodes recovered from specific lung cell types are sequenced, maps lipid structure to cell-type delivery rules directly in lung tissue.^53^ This direct tissue-to-design feedback speeds selection of chemistries that pair aerosol robustness or lung tropism with efficient cytosolic delivery, while general LNP design principles continue to guide escape kinetics, biodegradation, and repeat-dose tolerability.^20^

However, despite this progress, mRNA LNP platforms still present several safety liabilities. Complement-activation-related pseudoallergy can trigger acute infusion reactions, including flushing, dyspnea, or even anaphylaxis. Anti-PEG IgG/IgM raised after repeated dosing may accelerate blood clearance of PEGylated particles and increase the risk of hypersensitivity reactions.^58,59^ In addition, intramuscular or intravenous administration of mRNA LNP often leads to high hepatic accumulation, which can both reduce delivery efficiency and pose a risk of hepatotoxicity.^60,61^ The pronounced liver tropism of many ionizable LNP is thought to arise from apolipoprotein adsorption (e.g., ApoE) and subsequent uptake by hepatocytes and Kupffer cells via low-density lipoprotein receptor (LDLR) and scavenger pathways. Formulation variables, including ionizable lipid p*K*_a_ and headgroup chemistry, helper-lipid identity (e.g., cholesterol analogs, DSPC/DOPE balance), PEG-lipid mol % and chain length, particle size, and surface charge, jointly tune this process and also influence endosomal escape and the translation efficiency of the delivered mRNA. The attendant risk of hepatotoxicity likely reflects a combination of innate immune activation (pattern-recognition receptor signaling, complement activation), lipid metabolite stress, and high local dose exposure. The use of biodegradable ionizable lipids and optimized PEG content can mitigate but not fully eliminate these liabilities.^60,61^ To redirect exposure toward the lung, several nonexclusive strategies are being explored. First, rational tuning of LNP structure and composition can reduce ApoE-mediated hepatic uptake and bias deposition to the pulmonary microvasculature or airway epithelium.^55^ Second, introducing amide and urea linkers into the ionizable-lipid scaffold can alter hydrogen-bonding networks, rigidity, and degradability, which can improve lung tropism while maintaining endosomal release, although linker stability must be balanced with timely cargo unpackaging.^56,57^ Additional levers include SORT designs using charged helper lipids, ligand decoration for cell-type specificity (e.g., endothelial versus alveolar epithelial cells), and the option of orthogonally changing the route of administration. Local pulmonary delivery via nebulized or dry-powder aerosols can bypass hepatic first-pass metabolism and reduce systemic exposure, but faces barriers including mucus and surfactant, mucociliary clearance, and uptake by alveolar macrophages; strategies such as mucus-penetrating coatings and aerosol-resilient particles are active areas of optimization.^12,13^ Key translational considerations are repeat-dose tolerability (e.g., anti-PEG responses), complement-activation-related pseudoallergy, lot-to-lot manufacturability at scale, and device compatibility for inhaled formats. Finally, accurate biodistribution quantitation via radiolabel or positron emission tomography imaging, capillary depletion assays, and single-cell transcriptomics, as well as optical tracking with biocompatible fluorescent quantum dots,^62,63^ will be essential to confirm true lung-cell transfection (not just vascular trapping) and to guide iterative LNP design for durable, safe pulmonary mRNA delivery.

Prolonged administration can also lead to lipid accumulation in hepatocytes, which has been linked to transient liver-enzyme elevations and, in some models, steatosis.^58,59,64^ Also, bronchospasm and surfactant disruption were identified as specific concerns for inhaled formulations, particularly those with high cationic-lipid content.^65^ To address these challenges, researchers are actively investigating next-generation biodegradable ionizable lipids that fragment into water-soluble metabolites, cleavable stealth coatings that shed within acidic endosomes, and stimuli-responsive polymers that disintegrate in hypoxic tumor microenvironments.^66^ In addition, a recent study showed that substituting PEG with high-density brush-shaped polymer lipids markedly reduces anti-PEG antibody binding and preserves protein expression after repeated mRNA dosing.^67^ Moreover, incorporating in mRNA nanocarriers cyclic-dinucleotide-mimetic lipids that activate the stimulator of interferon genes (STING) pathway may even turn the carrier itself into an adjuvant, converting every delivered transcript into a self-amplifying immune alarm.^68^

## Therapeutic mRNA Payloads

Therapeutic mRNA payloads for lung cancer now cover a wide range, but they all aim to deliver proteins that mark tumor cells for immune attack, reprogram the tumor microenvironment, or correct harmful cancer-related pathways (Figure 3). Over the past five years, the clinical portfolio has broadened from proof-of-concept vaccines to dose-escalation studies that test mRNA encoding cytokines, Kirsten rat sarcoma oncogene (KRAS) mutant and neoantigen constructs, bispecific “antibody factories,” tumor-suppressors, and even lung-tropic CRISPR systems. These investigations already include intramuscular, intradermal, subcutaneous, intravenous, and inhaled routes, highlighting the rapid integration of mRNA technology into early phase oncology trials (Table 1).

**Figure 3:**
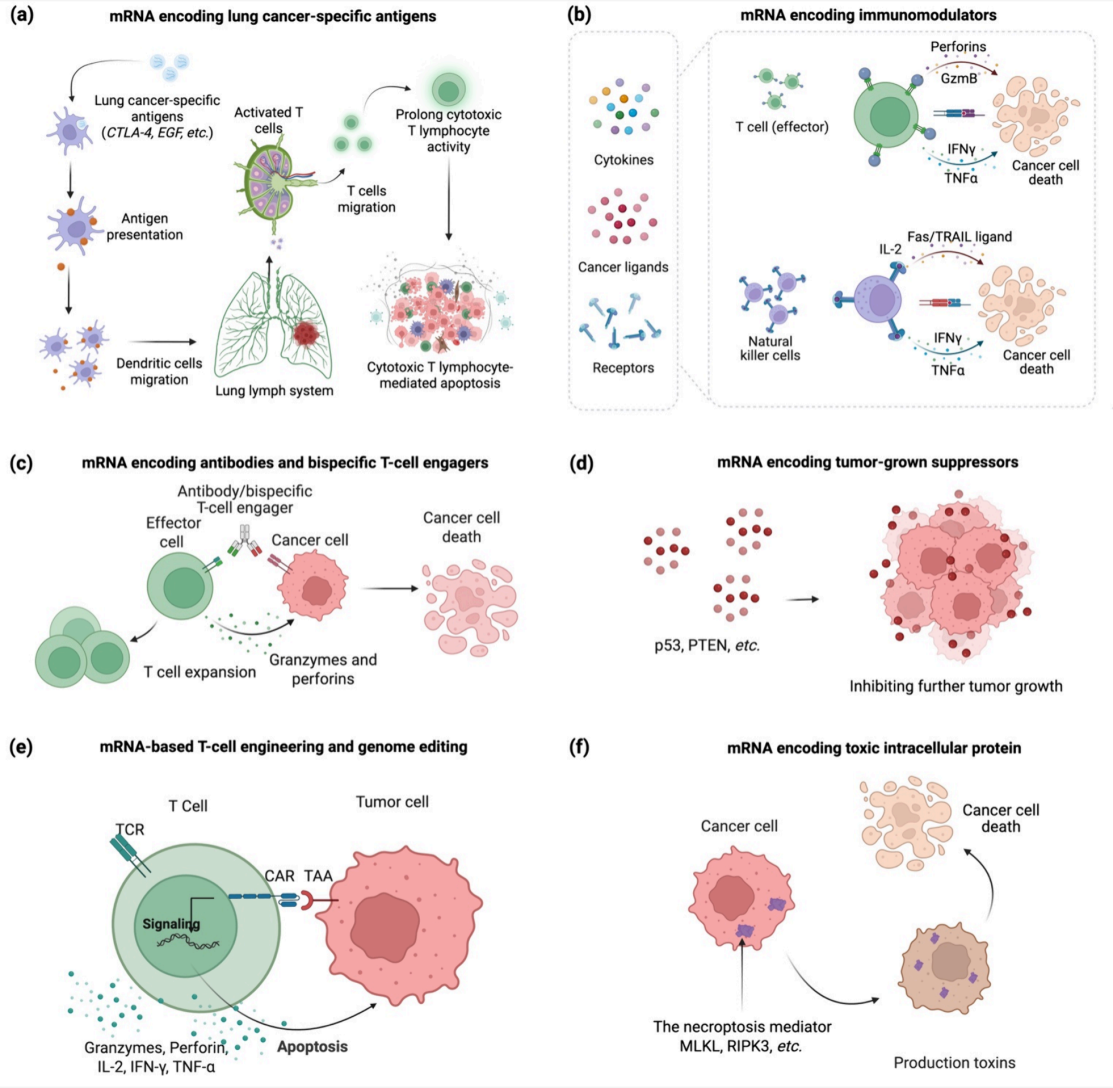
mRNA payloads for lung-cancer therapy. (a) mRNA encoding shared or patient-specific lung-cancer antigens sparks cytotoxic T-cell immunity. CTLA-4 – cytotoxic T-lymphocyte-associated protein 4; EGF – epidermal growth factor; (b) mRNA encoding immunomodulators reprogramme the tumor microenvironment and intensify by-stander immunity; GzmB – granzyme B; TNF-α – tumor necrosis factor alpha; (c) mRNA encoding antibody and T-cell-engager, which can convert host cells into transient producers of full-length IgG or bispecific T-cell engagers that amplify targeted killing; (d) mRNA encoding tumor-suppressors, which can reactivate growth-inhibitory pathways and resensitize resistant tumors; P53 – tumor protein p53; PTEN – phosphatase and tensin homologue; (e) T-cell engineering and genome editing – CD3- or lymphoid-tropic LNP deliver mRNA encoding CAR/TCR or Cas9 to program effector lymphocytes *in vivo* for selective tumor apoptosis; TCR – T-cell receptor; (f) mRNA encoding toxic intracellular effectors (e.g., mixed lineage kinase domain-like pseudokinase (MLKL), receptor-interacting protein kinase 3 (RIPK3), or other lethal proteins initiate intrinsic death) within cancer cells.

**Table 1:** Completed and Ongoing Clinical Trials of mRNA-Based Lung Cancer Therapies during the Last 5 Years, from 2020 to July 2025^a^

Indication	RNA therapeutics (administration type)	Company/University	Clinical Trials.gov identifier (phase)	Date and status	Purpose
Pulmonary Osteosarcoma	Tumor mRNA-LNP (*i*.*v*.)	University of Florida	NCT05660408 (I/II)	2024–2026 Active	Determination of MTD dose, EFS, and OS for mRNA-LP
Colorectal/Pancreatic/NSCLC	mRNA-5671/V941 (*i*.*m*.)	Merck Sharp and Dohme LLC	NCT03948763 (I)	2019–2022 Terminated (Business reasons)	Efficacy and dose determination of V941(mRNA-5671/V941) as a monotherapy and in combination with pembrolizumab infusion.
NSCLC/Esophageal cancer	mRNA vaccine encoding neoantigen (*s*.*c*.)	Stemirna Therapeutics	NCT03908671(N/A)	2019–2025 Recruiting	Evaluation of mRNA tumor vaccine
NSCLC	mRNA vaccine encoding neoantigen (N/A)	Guangdong Provincial People’s Hospital	NCT06735508 (I)	2025–2026 Not yet recruiting	The safety, ability, immunogenicity, and preliminary efficacy in combination with adebelimab
NSCLC	mRNA-BI 1361849 (*i*.*d*.)	Ludwig Institute for Cancer Research	NCT03164772 (I/II)	2017–2021 Competed	The safety and preliminary efficacy of the addition of a vaccine therapy to 1 or 2 checkpoint inhibitors
NSCLC	Personalized mRNA tumor vaccine RGL-270 (N/A)	Nanjing Tianyinshan Hospital	NCT06685653 (I)	2026–NA Not yet recruiting	The safety and tolerability of RGL-270 targeting tumor-specific neoantigens and adebrelimab
Recurrent pulmonary/unresectable osteosarcoma and pHGG	RNA-LNP vaccine (*i*.*v*.)	University of Florida	NCT05660408 (I/II)	2025–2035 Active	The manufacturing feasibility, safety and immunologic activity
Advanced lung cancer and lung metastasis of solid tumors	Antigen dry powder vaccine BMD006 (*inh*.)	Cancer Institute and Hospital, Chinese Academy of Medical Sciences	NCT06928922 (I)	2025–2028 Recruiting	The safety, tolerability, preliminary efficacy, PK, and PD + the effect of BMD006 in combination with PD-1 treatment
sqNSCLC	mRNA CV09070101 (CVHNLC) (*i*.*m*.)	CureVac	NCT07073183 (I)	2025–2029 Not yet recruiting	The safety and tolerability of CVHNLC with pembrolizumab, CVHNLC plus prembrolizumab and chemotherapy (carboplatin and paclitaxel)
KRAS mutant advanced or metastatic NSCLC, colorectal cancer or pancreatic adenocarcinoma	mRNA-5671/V941 (*i*.*m*.)	Merck Sharp and Dohme LLC	NCT03948763 (I)	2019–2021 Terminated (Business reasons)	The safety and tolerability of mRNA-5671/V941 as a monotherapy and in combination with pembrolizumab

a NSCLC – nonsmall cell lung cancer; sqNSCLC – squamous nonsmall-cell lung cancer; pHGG – pediatric high-grade gliomas; KRAS – Kirsten rat sarcoma oncogene; MTD – maximum tolerated dose; ORR – objective response rate; OS – overall survival; LNP – lipid nanoparticle; EFS – event-free survival; *i*.*v*. – intravenous injection; *i*.*m*. – intramuscular injection; *i*.*d*. – intradermal injection; *s*.*c*. – subcutaneous injection; *inh*. – inhalation; N/A – not applicable.

### Antigen-Encoding Vaccines

The most clinically advanced area in mRNA-based cancer therapy is vaccination employing tumor-associated or tumor-specific antigens (Figure 3a).^8^ Protamine-formulated CV9201 is an mRNA vaccine that encodes five shared NSCLC antigens, and its later version CV9202 (BI 1361849) includes the same five sequences plus a sixth, Mucin 1, within a single transcript. In a phase Ib trial in stage IV NSCLC, intradermal CV9202 combined with radiotherapy elicited broad immunogenicity, with most patients (84%) developing antigen-specific responses, including antibody production (80%), functional T-cell activity (40%), and reactivity against multiple antigens (52%).^34^ A follow-on phase I/II trial combining CV9202 with durvalumab ± tremelimumab (NCT03164772) has demonstrated good tolerability and T-cell activation in patients, but progression-free survival data have not yet been published.^34^ Whereas CV9202 delivered a predefined “antigen cassette”, the mRNA-5671/V941 vaccine developed by Merck encodes the four most prevalent driver KRAS mutations, namely, G12C, G12D, G12 V, and G13D, within a single LNP transcript. A phase I, first-in-human dose-escalation trial of mRNA-5671/V941 (NCT03948763) finished in 2022, showing robust CD8^+^ responses in patients, although objective-response or progression-free survival data have not been released yet.^8^ Looking ahead, BioNTech’s BNT116, an mRNA-lipoplex vaccine that encodes six nonmutated antigens frequently expressed in NSCLC (CLDN6, KK LC 1, MAGE A3, MAGE A4, MAGE C1, and PRAME), entered the phase I first-in-human LuCa-MERIT-1 trial (NCT05142189) in 2023. Preliminary data presented at the 2023 Society for Immunotherapy of Cancer Annual Meeting showed the regimen (BNT116 ± cemiplimab or docetaxel) was well tolerated but, in the monotherapy cohort, produced stable disease in 6 of 10 evaluable patients and no confirmed partial responses so far. Ongoing combination cohorts will test whether chemotherapy or programmed-cell-death-1 (PD-1) checkpoint blockade broadens antigen spread and amplifies vaccine efficacy.^69^

Taken together, these trials underscore key factors for future mRNA lung vaccines that will be related to (i) antigen selection, (ii) delivery and formulation, (iii) combination with radiotherapy and immunotherapy, and (iv) stratification by tumor type and biomarkers. For example, the first-generation vaccines targeted shared tumor-associated antigens (e.g., NY-ESO-1, MAGE, CEA, MUC1),^34^ but results suggest focusing on patient-specific neoantigens may be more effective. Modern trials increasingly use tumor sequencing to pick high-quality neoantigens for each patient.^70^ CureVac’s RNActive vaccines were protamine-formulated and given intradermally,^34^ whereas BioNTech/Moderna uses lipid-based carriers (LNP or RNA-lipoplexes). Also, the choice of delivery can affect immunogenicity and tolerability; indeed, comparisons suggest dendritic cell transfection (old approach) gave limited clinical benefit.^70^ Next-generation LNP systems are now favored for potent innate activation.^70^ The optimal administration routes are still under study (e.g., intradermal, intramuscular, and inhalation); systemic intravenous delivery remains experimental.^70^ Clinical trials combining mRNA vaccines with radiotherapy or checkpoint inhibitors have shown more encouraging outcomes. For instance, CV9202 combined with hypo-fractionated radiotherapy induced multiantigen T-cell responses and lung cancer control in nearly half of patients.^34^ Likewise, early data on vaccines plus anti-PD-1/-PD-L1 (e.g., cemiplimab, durvalumab, tremelimumab) show higher response rates than vaccine alone.^70^ These findings suggest future lung cancer vaccines should be given in combination regimens or with built-in adjuvants. Stratification by tumor type and biomarkers could also be important and was highlighted in a clinical trial of CV92027, where patients were stratified by NSCLC subtype (squamous versus nonsquamous) and EGFR-mutation status.^34,71^ The BNT116 trial specifically enrolled patients with NSCLC that frail with PD-L1 ≥ 1%, acknowledging that mRNA vaccines may work best in those already prone to immunogenic tumors. Going forward, clinical trials could likely require genomic and immunologic profiling (e.g., tumor mutational burden, human leukocyte antigen type) to match the right antigen payload to each patient.^70^ Thereby, completed NSCLC mRNA vaccine trials showed that these therapies are generally safe and can elicit broad immune responses,^18,34^ but monotherapy activity has been limited so far. Early terminations of KRAS-targeting and other studies emphasized the need for a careful design. Key optimization strategies include selecting highly immunogenic neoantigens (often personalized), improving delivery (e.g., optimized LNP formulations and routes), and combining vaccines with immune-modulatory therapies. With these lessons, ongoing trials (e.g., personalized neoantigen vaccines like Moderna’s V940/mRNA-4157 in adjuvant NSCLC) may yield more definitive results. Therefore, continued research should clarify how to maximize antigen expression, tailor patient selection, and harness mRNA vaccines’ innate advantages to achieve meaningful clinical benefits in lung cancer.^18,70^

### Immunomodulatory Cytokines and Costimulatory Ligands

mRNA opens a modular route to deliver intractable cytokines and costimulatory ligands directly into the lung tumor microenvironment (Figure 3b). For instance, systemic recombinant IL-12 can generate lethal cytokine-release syndromes, yet local expression drives robust TH1 polarization and tumor regression.^9^ A single intratumoral injection of *saIL-*12-mRNA-LNP eradicated large primary tumors in mice and protected against tumor rechallenge.^72^ Preliminary human data showed that MEDI1191, a single-chain *IL-*12*p*70-mRNA administered by direct injection into cutaneous or subcutaneous tumors, when combined with durvalumab, could induce a strong IFN-γ induction and T-cell infiltration without systemic toxicity.^8,73^ For deep lung lesions that are hard to access percutaneously, inhalable extracellular-vesicle formulations of *IL-*12-mRNA have achieved uniform alveolar deposition, regression of orthotopic LL/2 lung tumors, and abscopal immunity against distant metastases in mice.^22^ Moreover, *ex vivo* electroporation of adoptively transferred CD8^+^ T cells with *IL-*15-*sushi*-mRNA could prolong their persistence and augment cytotoxicity, and this technology also showed the possibility of *in vivo* transfection with T-cell-targeting LNP for lung cancer.^74^ In addition, costimulatory ligands such as OX40L, CD40L, or 4-1BBL encoded by mRNA have also proved capable of repartaterning suppressive tumor microenvironment, and the intratumoral mRNA-2416 encoding human OX40L showed a favorable safety profile in an early clinical trial (NCT03323398).

### Antibody, T-Cell Engagers, and Tumor Suppressors

Beyond vaccines and immunostimulants, synthetic mRNA can transiently convert the patient’s cells into “biologic factories” that secrete therapeutic antibodies inside or near the thoracic tumor (Figure 3c). This paradigm addresses two long-standing problems of recombinant monoclonal antibodies: manufacturing and short intratumoral half-life.^9^ Preclinical studies have already shown that intravenous injection of ≤10 μg mRNA encoding a full-length anti-PD-1 IgG (pembrolizumab) encapsulated in LNP could achieve serum exposures comparable to milligram doses of the recombinant antibody, while avoiding Fc-mediated immune-complex toxicities associated with protein therapy.^75^ Although the seminal experiment was performed in a colorectal model, the pharmacokinetics were systemic and could be relevant to lung cancer, where anti-PD-L1 antibodies have been considered as a first-line therapy. Furthermore, a single 10 μg dose of an mRNA encoding an EGFR × CD3 “LiTE^RNA^” bispecific T-cell engager eliminated established EGFR-positive tumors in mice and generated durable memory T-cell responses, illustrating the potential of this strategy to against EGFR-overexpressing NSCLC.^76^ In a phase I/II trial (NCT05262530), BioNTech’s BNT142 mRNA encoding CLDN6 × CD3 bispecific T-cell engagers (named RiboMab) is currently enrolling patients with cancers, including NSCLC. Meanwhile, preclinical development of a costimulatory CLDN6 × CD28 variant (SAR445197) is advancing through investigational new drug-enabling studies, providing a potential safety framework for future lung-targeted mRNA-encoding bispecific T-cell engagers.^77^

A complementary strategy to immune activation is to reinstall lost tumor-suppressor function (Figure 3d). *p*53 is mutated in ∼50% of lung adenocarcinomas and ∼65% of squamous tumors, while *PTEN* (*phosphatase and tensin homologue*) loss, *KEAP*1 (*Kelch-like ECH-associated protein* 1) inactivation, and *RB1* (*retinoblastoma transcriptional corepressor* 1) deletion are also common.^9^ Re-expressing wild-type p53 with redox-responsive LNP carrying *p53*-mRNA restored micromolar p53 protein, induced apoptosis, and resensitized *p*53-null NSCLC xenografts to everolimus, an mTOR inhibitor previously ineffective in *p53*-deficient settings.^78^ Systemic delivery of *PTEN*-mRNA nanoparticles reversed immune exclusion, up-regulated intratumoral IL-12 and TNF, and synergized with anti-PD-1 therapy to eradicate *PTEN*-null lung tumors in mice.^79^ Although no tumor-suppressor mRNA has yet entered the clinic, a GMP-grade p53 mRNA-LNP candidate is reported to be nearing investigational new drug submission for solid-tumor cohorts that include refractory NSCLC.^78,80^ The appeal of this modality is its mutation-agnostic design: rather than inhibiting one oncogenic driver, it simply repairs the master “guardian” gene itself.^78,81^

Conventional *ex vivo* CAR-T or TCR engineering is labor-intensive and often yields too few functional lymphocytes from elderly or heavily pretreated lung-cancer patients (Figure 3e). Proof-of-concept studies have demonstrated that systemic delivery of CD3-targeted, ionizable-lipid LNP encoding an anti-CD19 CAR can generate functional CAR-T cells *in vivo*. These *de novo* CAR-T cells eradicated disseminated lung metastases in mice and disappeared within a week as the mRNA degraded, thereby reducing the risk of prolonged cytokine release or on-target/off-tumor toxicity.^82^ Moreover, clinical genome-editing of T cells is already feasible: first-in-human Cas9-edited T cells bearing an NY-ESO-1 TCR showed acceptable safety and persistence in patients with refractory solid tumors.^83^ In parallel, lung-targeted SORT LNP have delivered Cas9 mRNA and sgRNA selectively to pulmonary tissue in rodents, achieving homology-directed repair without extra-pulmonary editing.^38^ Combining such lung-restricted mRNA-based genome editors with NY-ESO-1-style CRISPR-T cells could potentially enable *in situ* correction of tumor-intrinsic drivers of resistance to PD-1/PD-L1 checkpoint blockade and to KRAS-directed targeted therapy, such as *STK*11 or *KRAS* in advanced NSCLC.^42,84,85^ mRNA encoding death-executor proteins provides additional therapeutics that can destroy cancer cells while simultaneously sounding an “immunogenic-danger” alarm within the tumor (Figure 3f). For instance, mRNA LNP encoding MLKL (mixed lineage kinase domain-like pseudokinase),^86^ could provoke tumor necroptosis *in situ*, ^83^arrest tumor expansion, and expose neo-epitopes that convert the tumor microenvironment from “cold” into anti-PD-1-responsive. Nanostructured silica nanoparticles carrying *RIPK*3 (*receptor-interacting serine/threonine kinase 3*)-mRNA could induce necroptosis and immune cell infiltration within the tumor.^87^ mRNA LNP encoding the N-terminal domain of Gasdermin-B showed the pore-forming ability to induce pyroptotic in breast and melanoma tumors,^88^ which could be potentially employed for treating lung cancer.

## Challenges and Future Directions

To summarize, within just over a decade, nanotechnology-enabled mRNA therapeutics have evolved from benchtop to emerging and potentially effective solutions for lung cancer in the clinic. Their power lies in unparalleled programmability: a single mRNA scaffold can be rapidly used to express virtually therapeutic payloads (e.g., immunomodulatory cytokines, tumor-suppressors, cytotoxic proteins, gene-regulatory, etc.), while the lipid or polymer carrier can be tuned for lung targeting with efficient endosomal escape ability in preclinical studies. The vaccines such as CV9202, mRNA-5671/V941, and BNT116 have already shown potent T-cell immunity and have provoked objective tumor regressions in advanced NSCLC in the clinical trials.^34,69^

However, the challenges of clinical mRNA application still distinguish today’s early trials from routine bedside practice. First, intratumorally heterogeneity permits antigen-negative clones to escape immune pressure, so polyvalent or fully personalized neoantigen cocktails will be essential.^16,36^ Second, the most potent cytokines (e.g., IL-12, IL-15, 4-1BBL) can provoke systemic inflammatory toxicities, and spatially confined delivery via lung-targeting LNP or inhalable extracellular vesicles remains crucial.^22,45^ Additionally, exploring cytokine mRNA candidates that balance therapeutic potency with systemic toxicity is important. We recently demonstrated that IL-10-mRNA nanoparticles can induce robust immune surveillance across diverse preclinical tumor models while mitigating systemic toxicities, suggesting this strategy could potentially be applied to lung cancer treatment.^89^ Third, for tumor-suppressor mRNA or CRISPR editors, both editing frequency in heterogeneous tumors and genomewide fidelity must meet clinical thresholds. Although lung-targeting SORT LNP improve on-target editing,^38,42^ rigorous off-target profiling is still required, and endosomal escape remains a major bottleneck that must be addressed before first-in-human dosing.^23^ Finally, manufacturing speed is also a bottleneck: if sequencing-to-product timelines stay at weeks, aggressive lung tumors may progress before bespoke RNA cocktails are ready.

Fortunately, researchers have been exploring potential solutions to address the aforementioned challenges. For instance, the high-throughput enzymatic RNA synthesis and continuous-flow microfluidic LNP assembly have already cut lead times to a few days in preproduction runs, pointing toward just-in-time vaccine or payload manufacture.^5,13^ Also, shrinking timelines from weeks to days increasingly rely on augmented artificial intelligence (AI) that links sequence design, carrier discovery, and process control. AI models trained on massively parallel reporter assay-scale sequence-function data can propose cap, UTR, and coding configurations that raise translational output, dampen innate sensing, and remain compatible with GMP workflows; those proposals are then reconciled with established design rules for chemically modified mRNA and circRNA cassettes.^10,39^ Personalization builds on experience from individualized vaccines, where multiomic tumor profiling and HLA-presentation predictions guide neoantigen selection in NSCLC and align payload choice with observed immunogenicity.^36,70^ On the delivery side, pooled in vivo bar-coded libraries can generate dense structure-to-tropism maps directly in lung tissue; active-learning across these maps prioritizes ionizable-lipid scaffolds and assembly “recipes” that combine aerosol robustness, lung selectivity, and efficient endosomal escape.^13,20,23,53,89^ Process-level surrogate models for microfluidic LNP assembly and inhalable-powder preparation predict size, PDI, encapsulation, potency, and aerodynamic performance from controllable parameters, enabling closed-loop adjustments, faster QC release, and reliable scale-out to hospital pharmacies.^13,20,45,52^ Genome-editor design is following the same trajectory: guide selection and lung-SORT formulations are increasingly informed by delivery-aware models and lung-derived on/off-target data sets, aligning editing efficiency with safety.^38,85^ Together, these AI-enabled loops compress the design-to-batch timeline and stabilize lot quality, exactly what bespoke lung-directed mRNA cocktails require.

Moreover, to potentially overcome persistence limitations, current progress is likely to converge along the following reinforcing axes. (i) Novel mRNA architectures, such as saRNA, taRNA, and circRNA, are extending translation windows while lowering dose requirements.^9,37,90^ Additionally, rational mRNA sequence design (for example, encoding a “zwitterionic” peptide tail like the EKP sequence) can shield translated therapeutic proteins from immune clearance, prolonging their circulation half-life and thereby reducing the required dose frequency.^91^ (ii) Bar-coded lipid libraries, SORT chemistries, and pooled *in vivo* screens are yielding carriers with lung specificity.^38,42,53^ In parallel, inhalable nanoparticle systems are being engineered to maximize safe pulmonary deposition and minimize systemic exposure, further enhancing lung specificity.^48^ (iii) Combination regimens that weave mRNA vaccines with checkpoint blockade, tumor-suppressor mRNA with mTOR inhibition, or *in situ* CAR programming with oncolytic viruses promise synergies unattainable with any monotherapy.^43,74^ (iv) Finally, distributed point-of-care mRNA synthesis may ultimately empower hospital pharmacies to transform a tumor’s sequencing profile into a tailored poly neoantigen vaccine in a rapid time frame, redefining the pace of personalized cancer immunotherapy.^5,6,9,13^

Hopefully, mRNA therapeutics could migrate from salvage-line experiments to front-line standards within the next few decades, potentially reshaping the treatment of lung cancer. Realizing that potential will depend on collaborations among mRNA chemists, nanomaterial engineers, systems immunologists, and clinical oncologists, while the path to durable, life-extending clinical outcomes remains challenging but increasingly within reach.
